# The influence of topical anesthetic and fluorescein on non-contact tonometry measurements using ultra-high-speed dynamic Scheimpflug

**DOI:** 10.1038/s41598-023-45165-5

**Published:** 2023-10-19

**Authors:** Marcelo Macedo, Marcelo Hatanaka, Wilma Lelis Barboza, Gabriella Marranghello Mingione, Renato Ambrósio, Remo Susanna

**Affiliations:** 1 Department of Ophthalmology, University of São Paulo, São Paulo, Brazil; 2 Department of Ophthalmology, Federal University of the State of Rio de Janeiro, Rio de Janeiro, Brazil

**Keywords:** Biophysics, Medical research, Risk factors

## Abstract

This study aimed to investigate the effects of topical anesthetic and fluorescein drops on intraocular pressure (IOP), central corneal thickness (CCT) and biomechanical properties as measured by Corvis ST (CST-Oculus; Wezlar, Germany) in healthy eyes. A cross-sectional observational study was conducted on 46 healthy patients. The CST measurements were obtained before and immediately after the instillation of topical anesthetic and fluorescein drops. Pre-post instillation data were statistically analyzed. IOP measurements were compared to Goldmann's Applanation Tonometry (GAT), which was also performed after drops instillation. Biomechanical parameters analyzed included applanation 1 velocity, applanation 2 velocity, applanation 1 time, applanation 2 time, whole eye movement, deflection amplitude, and stiffness parameter at first applanation. A statistically significant difference in IOP, both for non-corrected IOP (IOPnct) and biomechanically corrected IOP (bIOP), was observed before and after the instillation of eyedrops. Despite this statistical significance, the observed difference lacked clinical relevance. The IOPnct demonstrated a significant difference pre and post-anesthetic and fluorescein instillation compared to GAT (14.99 ± 2.27 mmHg pre-instillation and 14.62 ± 2.50 mmHg post-instillation, versus 13.98 ± 2.04 mmHg, with p-values of 0.0014 and 0.0490, respectively). Comparable findings were noted when justaposing bIOP to GAT (14.53 ± 2.10 mmHg pre-instillation and 13.15 ± 2.25 mmHg post-instillation, against 13.98 ± 2.04 mmHg, with p-values of 0.0391 and 0.0022, respectively). Additionally, CCT measurements revealed a statistically significant elevation following the administration of topical anesthetic and fluorescein drops (from 544.64 ± 39.85 µm to 586.74 ± 41.71 µm, p < 0.01. None of the analyzed biomechanical parameters showed statistically significant differences after drops instillation. While the administration of topical anesthetic and fluorescein drops induced a statistically significant alteration in both IOPnct and bIOP readings, these changes were not clinically consequential. Furthermore, a notable statistical rise was observed in CCT measurements post-drops instillation, as determined by CST. Yet, corneal biomechanical parameters remained unaffected.

## Introduction

Elevated intraocular pressure (IOP) is the primary risk factor associated with the development and progression of glaucoma, the leading cause of irreversible blindness worldwide^1,2^. Therefore, accurate measurement of IOP is crucial for diagnosing, monitoring, and controlling the disease. Despite its initial description in 1957, the Goldmann applanation tonometer (GAT) remains the "gold standard" technique for IOP measurement^3,4^.

Although GAT is commonly used in clinical practice, it is recognized to potentially underestimate IOP in eyes with thin corneas and overestimate in thicker ones^5–7^. Furthermore, biomechanical properties can also impact GAT IOP measurements^5^. Each eye has its unique biomechanical property, with variations in corneal and scleral thickness and elasticity between individuals and even within the same person. Additionally, the presence of corneal scars or irregularities can alter the anatomy, thickness, and biomechanical properties of the eye^5–7^. Consequently, the evaluation of glaucoma suspects or glaucoma patients is at risk of being interpreted incorrectly^8–10^.

It is now becoming more apparent that the impact of the cornea on the GAT measurement of IOP extends beyond just corneal thickness. Numerous studies have indicated that besides elevated IOP, thin cornea, age, and family history, the biomechanical properties of the cornea are also a significant independent risk factor for glaucoma^11–19^.

Eyedrops, including topical anesthetics and fluorescein, are commonly used in clinical situations such as corneal evaluations, diagnostic testing for ocular surface diseases, and IOP measurements with GAT. Previous reports have compared GAT before and after anesthetic and fluorescein instillation (nGAT and fGAT, respectively), and found that nGAT resulted in lower IOP measurements^20,21^. However, measurement techniques used in these studies were not compared to other devices that offer IOP measures adjusted for corneal thickness and biomechanical properties, such as Corvis ST (CST-Oculus; Wetzlar, Germany), which assesses corneal deformation in vivo ^22^.

The CST device provides a variety of measurements, including standard IOP (IOPnct), central corneal thickness (CCT), corneal biomechanical parameters, and a newly validated estimate of the corrected IOP (bIOP), which aims to eliminate the effects of corneal thickness and stiffness parameters on the measurement. Additionally, this non-contact device eliminates the need for eyedrop instillation during measurement^22–24^. The algorithm for determining the bIOP value was based on numerical simulations of dynamic corneal deformation. It takes into account asphericity, CCT, age-related changes in corneal rigidity and many other parameters^25,26^.

This study investigates the potential interference of topical anesthetic and fluorescein drops on CCT, corneal biomechanical properties and IOP measurements obtained with CST. Also, IOP measurements were compared to fGAT IOP.

## Methods

This cross-sectional observational study evaluated 46 eyes from 46 patients, including 23 right eyes and 23 left eyes chosen randomly. All participants had no ocular pathology and were seen at a single São Paulo, Brazil center. The study protocol was approved by the University of São Paulo's institutional review committee, adhered to the principles of the Declaration of Helsinki and informed consent was obtained from all subjects.

The study enrolled healthy participants aged 17 years or older who consented. Participants were excluded in the presence of ocular hypertension, glaucoma, prior eye surgery or inflammation, corneal abnormalities affecting IOP measurement with GAT and CST, continuos contact lens use, spherical refraction exceeding 5.00 diopters, corneal astigmatism greater than − 3.00 diopters, inability to maintain fixation and visual acuity worse than 0.3 (20/40).

All participants underwent comprehensive ophthalmic evaluations that included a medical history review, assessment of best-corrected visual acuity, slit-lamp biomicroscopy, and fundus examination. Additionally, IOP was measured by two types of tonometers, GAT (Haag-Streit International Con., Ltd, Koeniz, Switzerland) and CST air-puff. Through CST device, corneal deformation was induced while a high-speed camera with Scheimpflug geometry captured over 4300 frames per second, resulting in 140 images of the horizontal corneal meridian during the 30-ms air puff^27^.

The same trained nursing technician used CST to measure IOP of each eye sequentially, starting with the right eye and then proceeding to the left. Measurements obtained without anesthetic and fluorescein instillation were IOPnct (nIOPnct), bIOP (nbIOP), CCT and other CST variables. Subsequently, a drop of topical anesthetic (Anestalcon, Alcon, Brazil) followed by a drop of 1% fluorescein solution (10 mg/ml, Allergan, Brazil) were instilled in the lower conjunctival fornix and measurements were repeated using CST. The collected data after eye drops instillation included non-corrected IOP (fIOPnct), biomechanically corrected IOP (fbIOP), CCT and the same CST variables as before.

Following the instillation of eyedrops and obtaining measurements using CST, GAT was performed on all participants. GAT was conducted by the same experienced ophthalmologist (MM) using a properly calibrated tonometer in the same slit lamp. To prevent infection, the applanation tip was cleaned with 70% alcohol before each exam, removing any residual fluorescein dye.

CST analysis software version 1.6r2187a offers various parameters, including applanation 1 velocity (A1V), applanation 2 velocity (A2V), applanation 1 time (A1T), applanation 2 time (A2T), whole eye movement (WEM), deflection amplitude (DefA) and stiffness parameter at first applanation (SP-A1), all of which were analyzed in the present study and are described in Table 1.

**Table 1:** Description of CST parameters analyzed.

CORVIS ST parameters	Description
Applanation 1 velocity (A1V)	Speed of the corneal apex during the inward applanation
Applanation 2 velocity (A2V)	Speed of the corneal apex during the second applanation
Applanation 1 time (A1T)	Time from the air-puff beginning to the first inward applanation
Applanation 2 time (A2T)	Time of the outward applanation
Whole eye movement (WEM)	Posterior direction of the globe during air puff tonometer
Deflection amplitude	Difference between the WEM and the corneal apex displacement at the HC position
Stiffness parameter at first applanation (SP-A1)	Corneal stiffness related to maximum deformation between the corneal apex at the beginning of the examination to the moment of the first applanation

### Statistical analyses

Statistical analysis was performed on the data using the STATA 14.0 program (StataCorp LP, College Station, TX, USA). Descriptive analyses were conducted using frequency tables. The Wilcoxon Test was used to compare pre- and post-instillation measurements of anesthetic and fluorescein for variables such as IOPnct, bIOP and CCT. Bland–Altman was employed for concordance analysis to compare IOP measurements and CCT with and without the use of topical anesthetic and fluorescein drops. Multiple linear regression was used to investigate biomechanical factors associated with differences in pre- and post- eyedrops instillation measurements. IOP measured by CST and by GAT were also compared using the Kruskal–Wallis test and Dunn's post hoc analyses. Spearman's correlation was used to investigate the correlation between continuous variables of interest, as CCT and IOP measurements. A p-value less than or equal to 0.05 was considered statistically significant for all tests.

## Results

In the present study, 46 participants between the ages of 18 and 67 (mean 40.59 ± 12.48) were included, of which 58.7% were female. For statistical analysis, one eye from each patient was chosen randomly.

Using the Kruskal–Wallis test and Dunn’s post hoc analysis, significant statistical differences were observed in IOP measurements between nIOPnct and fGAT (14.99 ± 2.27 mmHg vs. 13.98 ± 2.04 mmHg, p = 0.0490) as well as between fIOPnct and fGAT (14.62 ± 2.50 mmHg vs. 13.98 ± 2.04 mmHg, p = 0.0014). In addition, the same was observed in IOP measurements between nbIOP and fGAT (14.53 ± 2.10 mmHg vs. 13.98 ± 2.04 mmHg, p = 0.0022) and between fbIOP and fGAT (13.15 ± 2.25 mmHg vs. 13.98 ± 2.04 mmHg, p = 0.0391), as shown in Table 2.

**Table 2:** Kruskal–Wallis test and Dunn's post hoc analysis results comparing between CST measures versus fGAT.

CST measures		fGAT	p value
nIOPnct	14.99 ± 2.27 mmHg	13.98 ± 2.04 mmHg	p = 0.0490
fIOPnct	14.62 ± 2.50 mmHg		p = 0.0014
nbIOP	14.53 ± 2.10 mmHg		p = 0.0022
fbIOP	13.15 ± 2.25 mmHg		p = 0.0391

There was a significant statistical difference in IOPnct measurements from CST before and after anesthetic and fluorescein instillation, with mean values of 14.99 ± 2.27 mmHg for nIOPnct and 14.62 ± 2.50 mmHg for fIOPnct (p = 0.0135). Moreover, there was a significant statistical difference between nbIOP (14.53 ± 2.10 mmHg) and fbIOP (13.15 ± 2.25 mmHg) measurements (p < 0.001).

Concerning CCT measurements, Wilcoxon test showed a statistically significant difference (p < 0.0001) between CST measurements before and after topical anesthetic and fluorescein instillation, with values of 544.64 ± 39.85 µm and 586.74 ± 41.71 µm, respectively (Table 3).

**Table 3:** IOP, bIOP, pachymetry and GAT measures before and after fluorescein instillation assessed by Wilcoxon Test.

	Before (mean ± sd)	After (mean ± sd)	p-value
IOP	14.99 ± 2.27	14.62 ± 2.50	0.0135
bIOP	14.53 ± 2.10	13.15 ± 2.25	< 0.0001
Pachymetry	544.64 ± 39.85	586.74 ± 41.71	< 0.0001
GAT	–	13.98 ± 2.04	

Spearman’s correlation analysis assessed the relationship between CCT and IOP measurements before and after anesthetic and fluorescein instillation. Statistically significant associations were considered when p ≤ 0,05. Analyzed variables and their respective p-values are listed in Table 4.

**Table 4:** Spearman’s Correlation analysis between CCT and IOP before and after eyedrops instillation with their corresponding p-values. Significant values are in bold.

	Spearman’s correlation	p-value
nIOPnct	0.1793	0.0872
fIOPnct	0.2181	**0.0367**
nbIOP	− 0.2699	**0.0093**
fbiop	− 0.2531	**0.0149**
nGAT	0.1828	0.0812
fGAT	0.1900	0.0697

**Figure 1:**
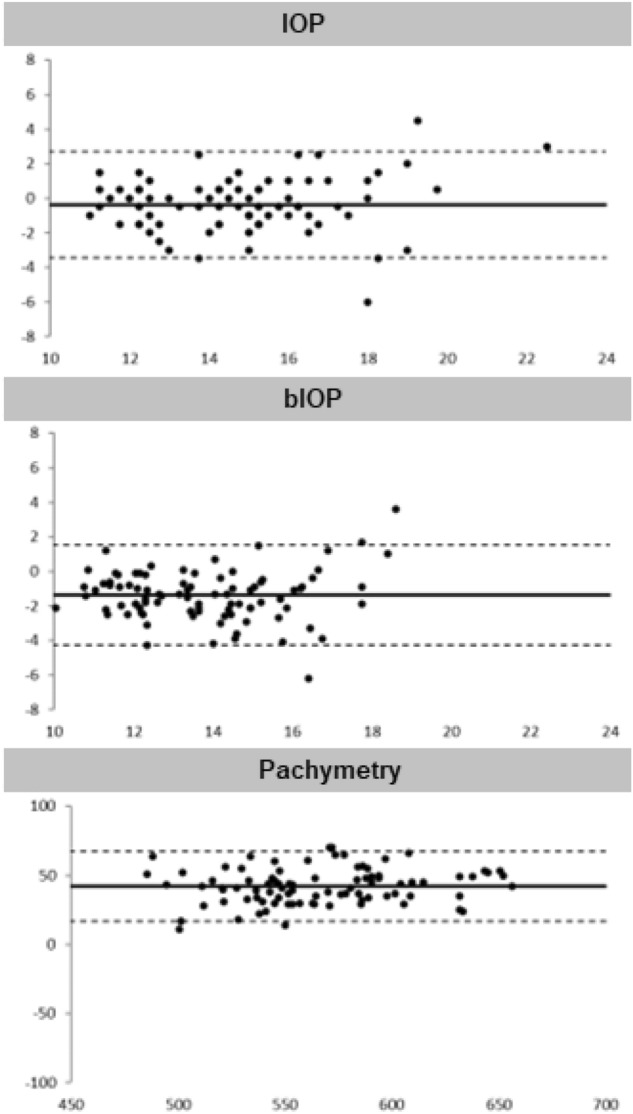
Bland–Altman plots before and after anesthetic and fluorescein instillation.

Figure 1 presents the Bland–Altman plots, which illustrate the variations in IOPnct, bIOP, and CCT measurements with and without topical anesthetic and fluorescein drops. Most of the measures recorded fell within the limits of agreement, indicating a low level of variability between the values. Nonetheless, the CCT plot shows poor agreement among the measurements.

Regarding the biomechanical parameters analyzed, the multiple linear regression revealed no statistically significant difference in values between pre- and post-eyedrops instillation conditions. All parameters presented p-values greater than 0.05, as shown in Table 5.

**Table 5:** Results of the Multiple linear regression analysis conducted on CST parameters along with their corresponding p-values.

CST parameters	P value
A1T	0.4590
A2T	0.9985
A1V	0.5676
A2V	0.5176
WEM	0.6958
DefA	0.8975
SP-A1	0.3107

## Discussion

The primary objective of glaucoma treatment is to halt the progression of optic nerve damage. Among the various risk factors associated with glaucoma, IOP is the only modifiable factor that can be targeted for treatment. Therefore, accurate measurement of IOP is imperative. The advent of GAT has facilitated studies exploring potential sources of IOP measurement fluctuations, including the Valsalva maneuver, eyelid squeezing, successive applanation tonometry and fluorescein quenching^28^. These investigations are particularly crucial due to the need for anesthetic and fluorescein eyedrops to perform IOP measurements using GAT.

**Figure 2:**
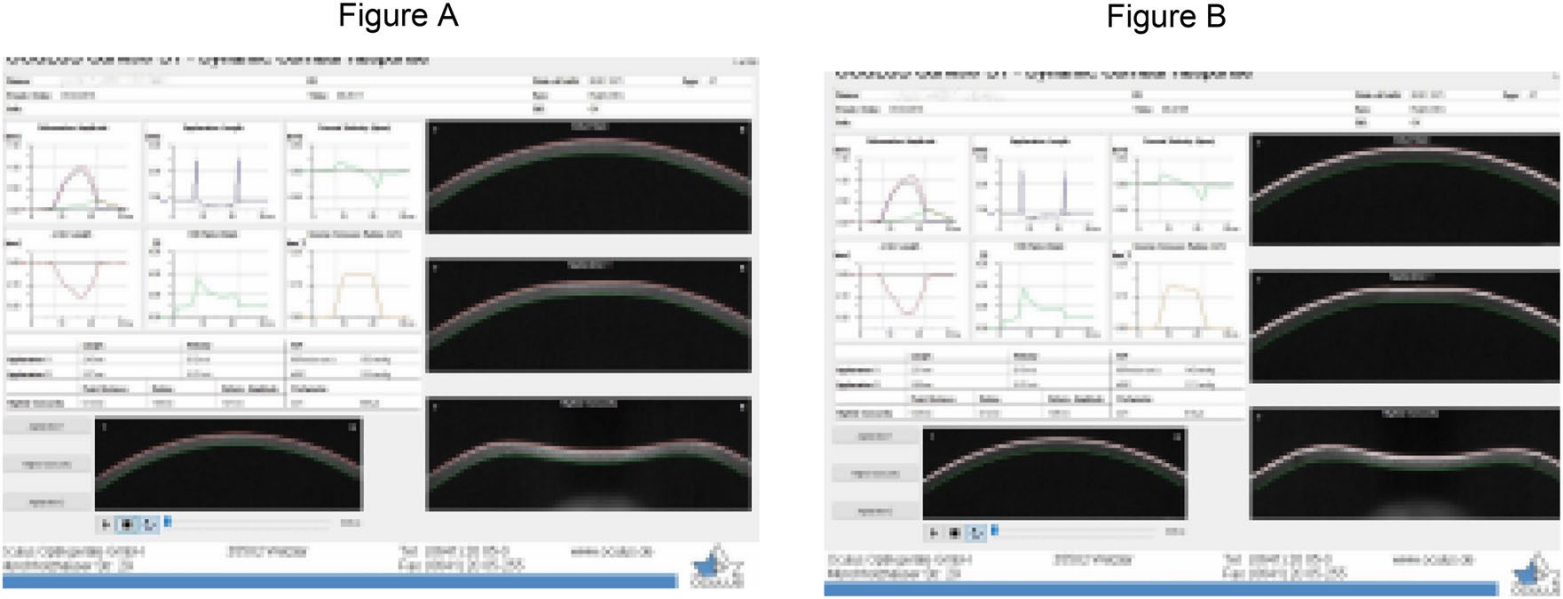
CST display capturing dynamic corneal response tab, comparing fluorescein-free (**A**) and fluorescein-stained (**B**) conditions.

Rosenstock et al.^29^ found that measurements conducted without fluorescein instillation may result in underestimation. Furthermore, both low and high fluorescein concentrations can lead to underestimation and overestimation of IOP^30–32^. Moreover, the use of anesthetic after fluorescein instillation can dilute it, leading to inaccurate IOP measurements^32^. However, none of these studies have examined the relevance of measurements with and without fluorescein instillation, along with its potential interference, and compared GAT to other measurement methods. In this study, we employed Scheimpflug geometry to record corneal surface images with and without fluorescein, investigating their association with IOP measurement and CCT (Fig. 2).

Our study observed a significant statistical difference (p < 0.001) when comparing CCT measurements before and after fluorescein administration. After eyedrops instillation, CCT measurements were approximately 40 µm higher than the baseline, which poses a challenge in obtaining precise IOP values when using a device that does not correct for external interference. This finding holds clinical relevance as previous studies have demonstrated that a 40 µm decrease in CCT elevates the risk of glaucoma progression^19,33^. Several papers have investigated the impact of CCT on GAT measurements^6,9^ and speculated that thin corneas might result in low IOP readings^10^, which is recognized as a risk factor for glaucoma progression^19^.

Besides CCT interference on the precision of GAT measurements, the biomechanical properties of corneal hysteresis (CH) can also play a role in it. Previous studies have indicated that CH may be a risk factor for glaucoma^19,34,35^ and additional biomechanical factors have been linked to the development and progression of the disease^16,17,36,37^. As a result, the advent of new technologies, like the Ocular Response Analyzer (ORA—Reichert Ophthalmic Instruments, NY, USA) and CST, is crucial for understanding the relationship between biomechanical properties and IOP^19,34,35^.

The biomechanical parameters obtained from CST were selected due to their association with the deformability of ocular tissues and susceptibility to pressure-induced harm. However, when examining these biomechanical parameters and their impact on bIOP, no statistically significant differences were observed.

Biomechanical parameters assessed in this study using CST are closely linked to alterations in IOP, meaning that an elevation in IOP would consequently impact these parameters ^15^. Although a statistically significant change in IOP was characterized by minor fluctuations that were not clinically relevant, no corresponding changes were observed in the analyzed parameters. Despite the documented variations in IOP, the biomechanical characteristics measured by CST remained stable and unaffected by these minimal IOP fluctuations. Analyzed biomechanical parameters are also part of those found by Serbecic et al.^38^ and by Bak-Nielsen et al.^39^ that demonstrated good repeatability and reproducibility. Good reliability for IOP and CCT measurements was also found ^38,39^.

In light of Spearman's correlation findings analyzing the relationship between CCT and IOP before and after anesthetic and fluorescein instillation, it was noted that higher pachymetry corresponded to higher IOPnct values but lower bIOP values. These results indicate that IOPnct is dependent on and affected by corneal thickness, unlike bIOP in which the increase in CCT induced by eyedrops instillation is accounted for, resulting in an IOP measurement less susceptible to corneal influences. These findings are consistent with Matsuura et al.’s findings ^24^.

Despite the statistically significant difference observed between bIOP and IOPnct when compared to fGAT in this study, the clinical relevance of these findings may be questioned due to GAT measurements limitations, which are expressed in whole or tens units without distinguishing decimal points, unlike CST. Therefore, incorporating these results into clinical practice may be challenging.

Although GAT remains the gold standard tool in IOP measurement, it's pivotal to acknowledge that no singular method is devoid of limitations. Both GAT and CST provide an estimated IOP, influenced differently by corneal properties. CST's approach aims to consider the dynamic behavior of the cornea, possibly making it more sensitive to certain biomechanical characteristics not explicitly accounted for in GAT^27^. Besides, it's also relevant to remember that the action of flattening the tonometer on the eye's surface during GAT measurement might temporarily alter the eye's natural biomechanical state, potentially affecting the readings^6^ These differences might explain why, in healthy eyes, we observed a variation between GAT and CST measurements post eyedrops instillation.

Our study emphasized the significance of incorporating several factors such as the instillation of eyedrops, age, genetics and biomechanical properties, to obtain a more precise assessment of IOP, a crucial predictor of glaucoma progression. These measures are particularly valuable for individuals suspected of having or diagnosed with glaucoma^12,13,18,36,40,41^.

This study has a few limitations such as the absence of a control group using an ultrasonic pachymeter to measure CCT and compare it with the CST measurements; the sample limitation to healthy eyes; and the absence of a stratified analysis by age groups to understand if the effects of the eyedrops or the consistency between the measures vary significantly between these groups. Future research is recommended to replicate these comparisons in patients with glaucoma and individuals with ocular hypertension and to incorporate the assessment of age-related corneal biomechanical parameters and alongside CCT.

## Conclusion

The findings of this study indicate that the administration of eyedrops may influence CCT measurements obtained using CST. Regarding IOP measurements, despite statistical significance no clinical relevance was observed. None of the biomechanical parameters analyzed showed statistically significant differences when comparing before and after the instillation of eyedrops.

## Data Availability

The datasets used and/or analysed during the current study available from the corresponding author on reasonable request.
